# Biogenic Polyphosphate Nanoparticles from a Marine Cyanobacterium *Synechococcus* sp. PCC 7002: Production, Characterization, and Anti-Inflammatory Properties In Vitro

**DOI:** 10.3390/md16090322

**Published:** 2018-09-10

**Authors:** Guangxin Feng, Shiyuan Dong, Min Huang, Mingyong Zeng, Zunying Liu, Yuanhui Zhao, Haohao Wu

**Affiliations:** College of Food Science and Engineering, Ocean University of China, 5 Yushan Road, Qingdao 266003, Shandong Province, China; fengguangxin@stu.ouc.edu.cn (G.F.); dongshiyuan@ouc.edu.cn (S.D.); 17864278962@163.com (M.H.); mingyz@ouc.edu.cn (M.Z.); liuzunying@ouc.edu.cn (Z.L.); zhaoyuanhui@ouc.edu.cn (Y.Z.)

**Keywords:** polyphosphate nanoparticles, *Synechococcus* sp. PCC 7002, anti-inflammation, macrophages, Toll-like receptors

## Abstract

Probiotic-derived polyphosphates have attracted interest as potential therapeutic agents to improve intestinal health. The current study discovered the intracellular accumulation of polyphosphates in a marine cyanobacterium *Synechococcus* sp. PCC 7002 as nano-sized granules. The maximum accumulation of polyphosphates in *Synechococcus* sp. PCC 7002 was found at the late logarithmic growth phase when the medium contained 0.74 mM of KH_2_PO_4_, 11.76 mM of NaNO_3_, and 30.42 mM of Na_2_SO_4_. Biogenic polyphosphate nanoparticles (BPNPs) were obtained intact from the algae cells by hot water extraction, and were purified to remove the organic impurities by Sephadex G-100 gel filtration. By using 100 kDa ultrafiltration, BPNPs were fractionated into the larger and smaller populations with diameters ranging between 30–70 nm and 10–30 nm, respectively. 4′,6-diamidino-2-phenylindole fluorescence and orthophosphate production revealed that a minor portion of BPNPs (about 14–18%) were degraded during simulated gastrointestinal digestion. In vitro studies using lipopolysaccharide-activated RAW264.7 cells showed that BPNPs inhibited cyclooxygenase-2, inducible nitric oxide (NO) synthase expression, and the production of proinflammatory mediators, including NO, tumor necrosis factor-α, interleukin-6, and interleukin-1β through suppressing the Toll-like receptor 4/NF-κB signaling pathway. Overall, there is promise in the use of the marine cyanobacterium *Synechococcus* sp. PCC 7002 to produce BPNPs, an anti-inflammatory postbiotic.

## 1. Introduction

Inorganic polyphosphates (polyPs) are polyanionic linear polymers composed of three to several hundred high-energy phosphoanhydride-bonded orthophosphate residues. PolyPs are a kind of “molecular fossil” that probably even predate life itself, and is ubiquitously distributed in all three kingdoms of life [1]. PolyPs are usually accumulated in living cells as granular particles within a subcellular organelle termed as acidocalcisome (also called volutin, a metachromic granule, or a polyP body in some organisms) in the form of its calcium/magnesium salt, and the size of the polyP granules depends on an organism itself and the living environment [2,3,4]. A variety of biological functions, such as a reservoir of phosphate, an alternative energy supply, a detoxifier of metals, a buffer against alkali, a protein-stabilizing chaperone, and a regulator of stress responses, inflammation, blood clotting, and bone mineralization, have been linked to polyPs in bacteria, yeast, fungi, and animals [5,6].

Probiotics are live microorganisms exerting beneficial effects on host health when administered in adequate quantities. Several recent studies suggest that certain probiotic effects, such as an enhancement of gut barrier function, the inhibition of intestinal fibrosis, and the attenuation of colon cancer progression, are mediated by some metabolites of probiotics (so-called postbiotics) [7]. Postbiotics are a mild alternative to probiotics that could irritate already inflamed intestinal tissue with increased permeability [8]. PolyPs have been discovered to be a postbiotic; they exert anti-inflammatory, anti-fibrosis, and antitumor activities in the gut [9,10,11,12]. The heterotrophic production of polyPs, a kind of energy-rich compound, demands luxurious energy and carbon sources such as glucose and propionate [13]. Many marine cyanobacteria, which are autotrophic on natural sunlight and CO_2_, accumulate large amounts of polyP granules within cells to combat the recurrent phosphorus starvation in the ocean [14]. They seem to be promising “photobioreactors” to produce polyPs in an energy and freshwater-saving way.

*Synechococcus* sp. PCC 7002 (hereafter *Synechococcus* 7002) is a unicellular marine cyanobacterium with excellent potential for industrial applications [15,16]. *Synechococcus* 7002 can make use of high-light irradiation, which enables this strain to grow fast with a doubling time shorter than 3 h [17]. *Synechococcus* 7002 survives a wide range of salt concentrations and temperatures, and can live photoautotrophically, mixotrophically, or heterotrophically [18]. This strain also naturally captures foreign linear double-stranded DNA and integrates them into its own genome by homologous recombination [19]. This confers a great advantage to *Synechococcus* 7002 in genetic engineering.

The current research discovered that polyPs were accumulated in *Synechococcus* 7002 as polyP nanoparticles, here called biogenic polyphosphate nanoparticles (BPNPs). Nanomaterials are increasingly used to boost nutrient and drug bioavailability in medical, nutraceutical, and food applications. Thus, *Synechococcus* 7002 seem to be an excellent “cell factory” for the production of postbiotic polyPs. We optimized the fermentation conditions for the production of BPNPs by *Synechococcus* 7002, and also characterized the particle, digestibility, and anti-inflammatory properties of BPNPs.

## 2. Results and Discussion

### 2.1. Production of BPNPs

**Figure 1:**
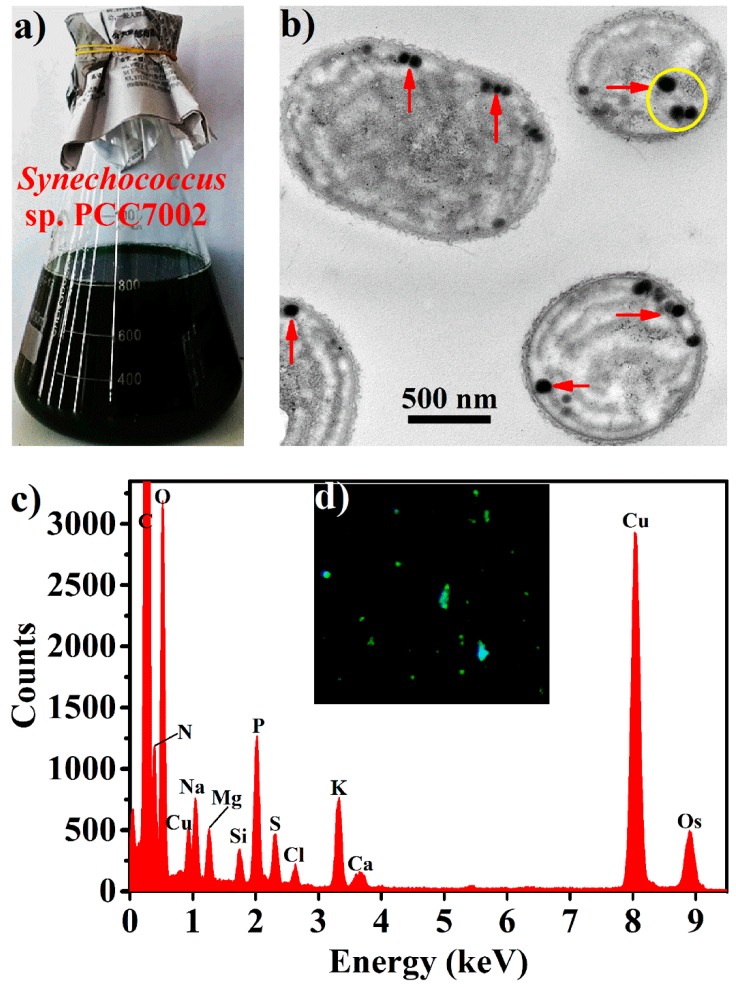
Characterization of biogenic polyphosphate nanoparticles (BPNPs) within the algae cells: (**a**) appearance of the *Synechococcus* 7002 culture, (**b**) typical transmission electron microscopic (TEM) image of thin sections of *Synechococcus* 7002 cells, (**c**) energy dispersive spectroscopy (EDS) analysis of the yellow circle region in panel b, and (**d**) fluorescence microscope image of *Synechococcus* 7002 cells stained with 4′,6-diamidino-2-phenylindole (DAPI, ×400).

*Synechococcus* 7002 cells (Figure 1a) contained near spherical electron-opaque granules with sizes typically smaller than 100 nm, according to transmission electron microscopic (TEM) observations of their thin sections (Figure 1b). Energy dispersive spectroscopy (EDS) of the yellow circle region in Figure 1b revealed that these granules contained abundant oxygen and phosphorus with the oxygen/phosphorus molar ratio calculated to be about three, corresponding to that in polyPs (Figure 1c). 4′,6-diamidino-2-phenylindole (DAPI) is frequently used to quantify and visualize polyPs due to its characteristic specific green fluorescence with polyPs [20]. As shown in Figure 1d, green fluorescent granules were observed within the DAPI-stained *Synechococcus* 7002 cells under light microscope. It thus seems that *Synechococcus* 7002 accumulated nanoparticulate polyPs (hereafter BPNPs) within cells.

PolyP granules have been observed prominently in many cyanobacterial cells such as *Nostoc pruniforme*, *Plectonema boryanum*, *Anabaena variabilis*, and *Synechococcus elongatus* PCC 7942 [21,22,23,24]. In these cells, they are relatively large with typical diameters of 200–400 nm. Interestingly, BPNPs in *Synechococcus* 7002 were much smaller than those in other reported cyanobacteria. This could probably be explained by *Synechococcus* 7002 being a rather smaller cyanobacterium with an average cell diameter of only 750 nm [25]. Particles smaller than 100 nm have been defined as nanoparticles. Compared with their bulk counterparts, nanoparticles usually display much higher biological activities due to their huge specific surface area and good bioavailability [26]. Therefore, BPNPs produced by *Synechococcus* 7002 are promising in future postbiotic applications.

**Figure 2:**
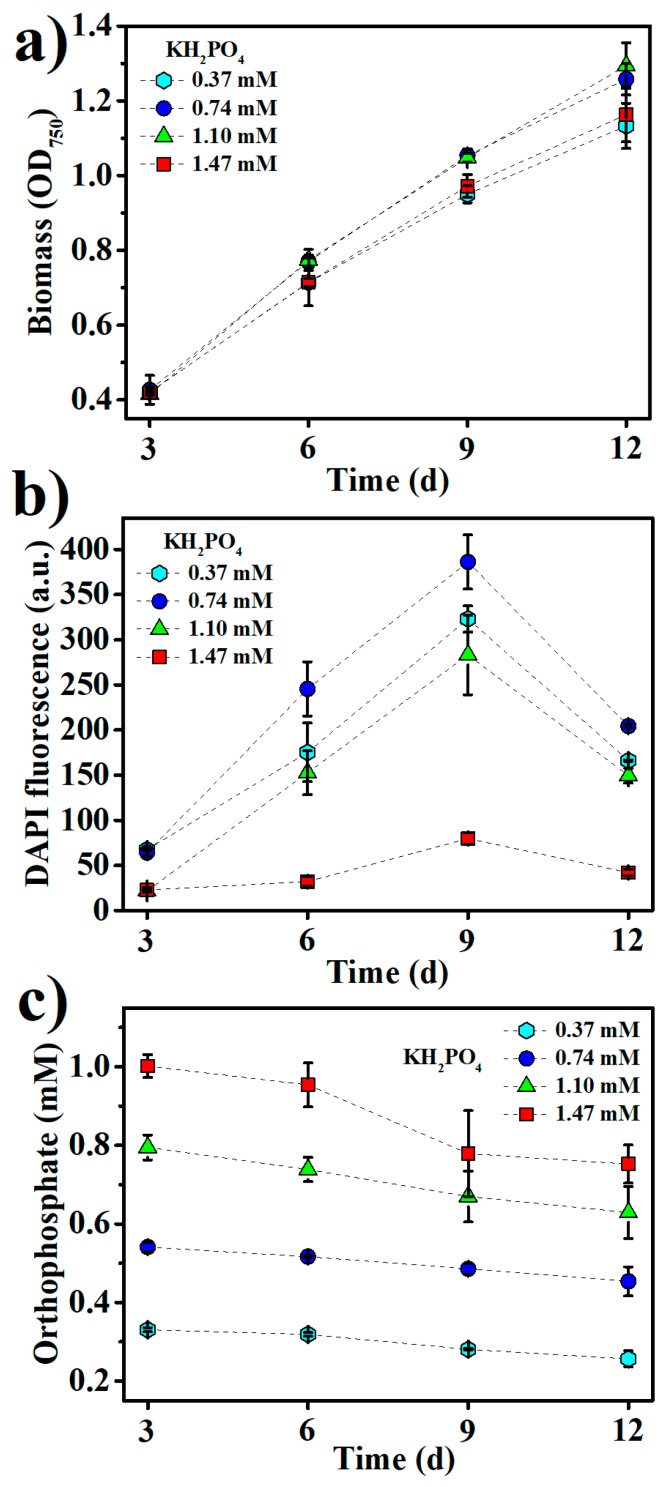
The kinetics of (**a**) biomass growth, (**b**) inorganic polyphosphates (polyP) accumulation, and (**c**) residual orthophosphate concentrations at various KH_2_PO_4_ concentrations in the medium.

In response to the fluctuating phosphorus supply in the ocean, polyPs have been used for the ample storage of phosphorus in many marine phytoplankton [14]. In this study, polyP accumulation in *Synechococcus* 7002 under different culture conditions was monitored by DAPI fluorescence. As shown in Figure 2a, the biomass of *Synechococcus* 7002 continued to increase within 12 days of growth, while polyP accumulation peaked at nine days of growth (late logarithmic phase), sharply declining thereafter (Figure 2b). This indicates that the high uptake of phosphorus by *Synechococcus* 7002 ceased in the late logarithmic growth phase. In regard of the high residual concentrations of phosphate in the mediums after nine days of growth (Figure 2c), phosphorus limitation is unlikely to be the reason for this cease of polyP accumulation, suggesting the growth phase dependence of polyP accumulation in *Synechococcus* 7002. In fact, the sharp decrease of polyP accumulation upon entering the stationary phase of growth has also been found in other microorganisms such as *Vibrio cholerae* and *Saccharomyces cerevisiae*, which is supposed to be due to the growth phase-dependent expression of several polyP-metabolizing enzymes such as polyphosphate kinase (PPK), exopolyphosphatase (PPX), and endopolyphosphatase (PPN) in microorganisms [27,28,29]. The microbial expression of PPX can be increased as large as 100-fold as growing cells approach the stationary phase, and polyP consumption seems essential for the stationary survival of microbial cells [30].

Initial KH_2_PO_4_ concentrations at 0.74 mM and 1.10 mM supported the better growth of *Synechococcus* 7002 than those at 0.37 mM and 1.47 mM (Figure 2a). The maximum polyP accumulation was found at 0.74 mM KH_2_PO_4_ in the medium (Figure 2b), and increasing KH_2_PO_4_ concentration above 0.74 mM decreased polyP accumulation, suggesting that much abundant phosphorus in the medium would adversely affect polyP accumulation. Under low phosphate concentrations, many bacteria use the phosphate (Pho) regulon to induce the expression of PPK to accumulate polyPs as a phosphorus reservoir, while high concentrations of environmental phosphate can negatively regulate the Pho regulon via a phosphate signal transduction protein, PhoU [31]. The Pho regulon has also been found to involve the phosphate homeostasis in *Synechococcus* sp. [32], so high KH_2_PO_4_ concentrations in the medium might negatively regulate the Pho regulon to downregulate polyP accumulation in this study.

**Figure 3:**
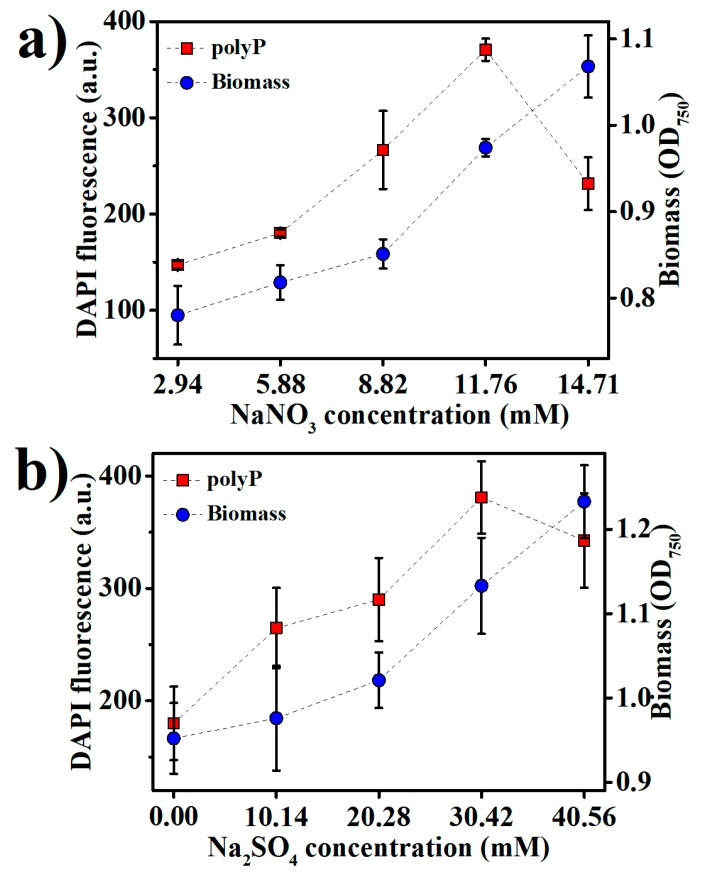
The accumulation of biomass and polyPs in *Synechococcus* 7002 after nine days of growth at various concentrations of (**a**) NaNO_3_ and (**b**) Na_2_SO_4_ in the medium.

PolyP storage is in fact a strategy in prokaryotes to cope with a variety of stresses, including the nutritional scarcities of not only phosphorus, but also several other nutrients, especially nitrogen and sulfur [33,34]. We examined the effects of nitrogen and sulfur levels in the medium on *Synechococcus* 7002 growth and polyP accumulation (Figure 3a,b). NaNO_3_ and Na_2_SO_4_ were added into the medium to adjust the nitrogen and sulfur contents, respectively. The optimal NaNO_3_ and Na_2_SO_4_ concentrations were 11.76 mM and 30.42 mM for polyP accumulation, respectively, while the best growth of *Synechococcus* 7002 apparently requires still higher concentrations of NaNO_3_ and Na_2_SO_4_ (Figure 3a,b), suggesting that the optimal nitrogen and sulfur contents for polyP accumulation were lower than those for *Synechococcus* 7002 growth. The sharply decreased polyP accumulation upon increasing NaNO_3_ and Na_2_SO_4_ concentrations over 11.76 mM and 30.42 mM, respectively, indicates that sufficient nitrogen and sulfur in the medium could retard polyP accumulation.

### 2.2. Purification and Characterization of BPNPs

**Figure 4:**
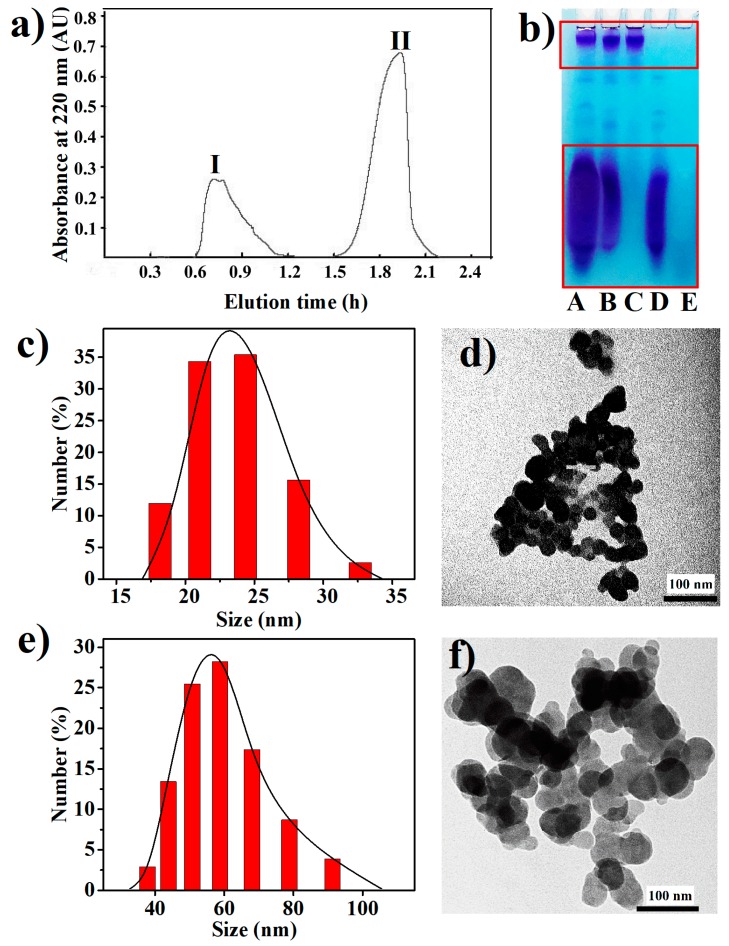
Purification and characterization of BPNPs: (**a**) purification of BPNPs from the cell extract of *Synechococcus* 7002 by Sephadex G-100 gel filtration, (**b**) the electrophoretic patterns for different fractions (A: the cell extract; B: peak I; C: >100 kDa fraction of peak I; D: <100 kDa fraction of peak I; E, peak II), (**c**,**e**) dynamic light scattering (DLS) size distributions and (**d**,**f**) TEM images of (**c**,**d**) larger BPNPs (L-BPNPs) and (**e**,**f**) smaller BPNPs (S-BPNPs).

The cell extract from *Synechococcus* 7002 was fractionated by gel filtration chromatography on a Sephadex G-100 column, and two peaks were pooled separately and named Fraction I and Fraction II, respectively (Figure 4a). The A, B, and E lanes in Figure 4b show an electrophoretic analysis of the cell extract, Fraction I and Fraction II, respectively. Two major separated bands were found in the A and B lanes, while no band was observed in the E lane. Apparently, BPNPs in the cell extract were eluted in peak I, which was the exclusion peak of Sephadex G-100 as located by using Blue Dextran (MW = 200,000) as a molecular weight marker (data not shown), while the organic impurities in the cell extract were eluted in peak II. This demonstrates that Sephadex G-100 gel filtration is an effective technique to purify BPNPs from the cell extract. In fact, polyPs are usually extracted from biological cells under acid or alkaline conditions, or with ethylenediaminetetraacetic acid (EDTA) at near neutral pH [1]. In this study, intact polyP nanoparticles were obtained from *Synechococcus* 7002 cells in a green way using hot water extraction followed by Sephadex G-100 gel filtration.

As shown in the C and D lanes of Figure 4b, the BPNPs in Fraction A could be further separated into larger and smaller fractions by 100-kDa ultrafiltration. Dynamic light scattering (DLS) was performed to determine the hydrodynamic sizes of BPNPs in the larger and smaller fractions. Figure 4c,e show the number size distributions of the larger BPNPs (L-BPNPs) and the smaller BPNPs (S-BPNPs), respectively. Single maximum peaks at 56 nm and 23 nm were observed for L-BPNPs and S-BPNPs, respectively. As shown in Figure 4d,f, L-BPNPs and S-BPNPs were visualized by TEM. Nearly spherical units with diameters in the ranges of 30–70 nm and 10–30 nm could be distinguished for L-BPNPs and S-BPNPs, respectively. The size of microbial polyP granules has been reported to be dependent on cell cycle stages, and smaller BPNPs can be found during cell division or the outgrowth of mature spores, probably because of the heavy consumption of phosphorus for nucleic acid synthesis at these stages [4,22,35]. Therefore, the L-BPNPs and S-BPNPs in this study were possibly derived from *Synechococcus* 7002 cells in different cell cycle phases.

### 2.3. Stability of BPNPs in Simulated Gastrointestinal Digestion (GID)

**Figure 5:**
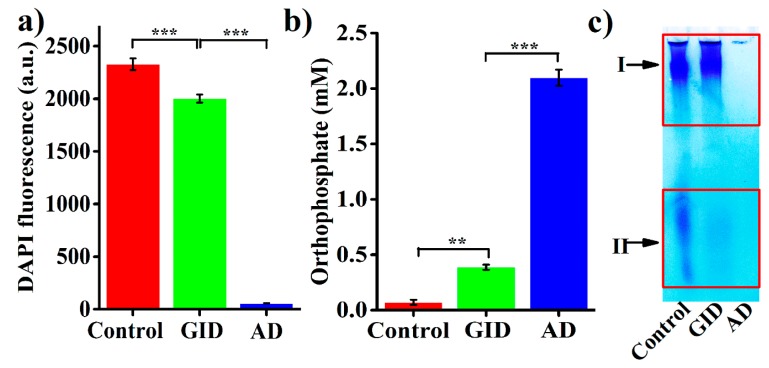
Stability tests of BPNPs in simulated gastrointestinal digestion (GID): (**a**) DAPI fluorescence, (**b**) orthophosphate production, and (**c**) gel electrophoretic analysis. Data were expressed as means ± standard deviations (*n* = 3). The complete hydrolysis of BPNPs into orthophosphate was achieved by acid digestion (AD) in 2 N HCl at 95 °C for 40 min. Statistical differences were determined by Student’s *t*-test (** *p* < 0.01, *** *p* < 0.001).

Considering their potential postbiotic applications, BPNPs were subjected to simulated GID to investigate whether they could reach intestinal epithelium in their intact form. The simulated GID resulted in a 14.1% reduction of the DAPI fluorescence of BPNPs (Figure 5a), and caused the significant production of orthophosphate (accounting for 18.4% of the total orthophosphate generated from the full hydrolysis of BPNPs) (Figure 5b). These results suggest that BPNPs were partially hydrolyzed under simulated GID. Figure 5c shows the results of a gel electrophoretic analysis of BPNPs before and after simulated GID. The band intensity at position II decreased markedly following the simulated GID, indicating that S-BPNPs could be degraded to a large extent during GID. However, no distinguishable reduction in the band intensity at position I was observed after simulated GID, suggesting that L-BPNPs could well survive GID.

The hydrolysis of polyPs is rather slow in neutral aqueous media at 25 °C, but its rate will rise sharply under acidic pH [1], possibly because hydrogen ions can neutralize the negative charges of phosphate residues, thereby facilitating the nucleophilic attack by water molecules during polyPs hydrolysis [36]. The enzymic hydrolysis of polyPs with *n* ≥ 5 in the gastrointestinal tract has been reported to be negligible [37]. Thus, it seems that the gastric acid-catalyzed hydrolysis of polyPs accounted for the GID-induced partial degradation of BPNPs. However, according to the results of DAPI fluorescence and orthophosphate production (Figure 5a,b), only a minor portion of BPNPs (about 14–18%) was degraded during simulated GID. Thus, BPNPs seem to survive the gastrointestinal digestion, which makes it possible to use BPNPs in therapeutic applications as a postbiotic nanomedicine.

### 2.4. Anti-Inflammatory Effects of BPNPs in Lipopolysaccharide (LPS)-Activated RAW264.7 Cells

**Figure 6:**
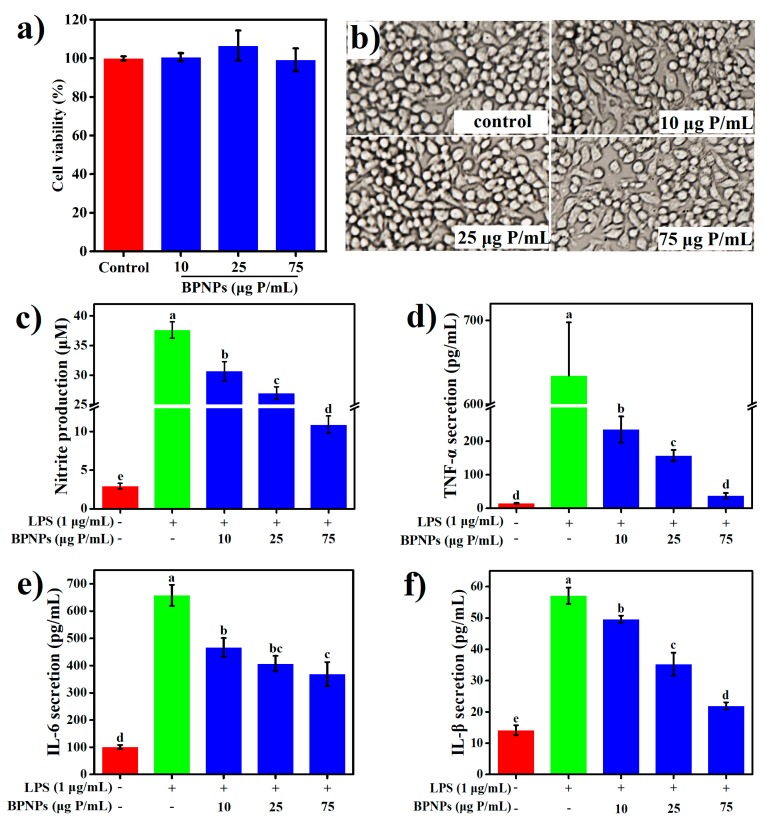
Effects of BPNPs on (**a**) survivability, (**b**) morphological appearance (20×), and production of (**c**) nitric oxide (NO) (*n* = 6), (**d**) tumor necrosis factor-α (TNF-α) (*n* = 3), (**e**) interleukin-6 (IL-6) (*n* = 3), and (**f**) interleukin-1β (IL-1β) (*n* = 3) in RAW264.7 cells following lipopolysaccharide (LPS) stimulation. Data were expressed as means ± standard deviations, with different lowercase letters marking significant differences (*p* < 0.05, one-way analysis of variance (ANOVA) followed by Turkey’s Honest Significant Difference (HSD) test).

LPS is a primary bacterial toxin initiating the inflammatory cascade during inflammatory bowel diseases (IBD). To evaluate the anti-inflammatory properties of BPNPs, RAW264.7 cells were pretreated with 10 μg, 25 μg, and 75 μg phosphorus (P)/mL of the particles for 6 h before stimulation with 1 μg /mL LPS for an additional 24 h. The results of methylthiazolyldiphenyl-tetrazolium bromide (MTT) assay (Figure 6a) and the morphological observation of cells with bright-field microscopy (Figure 6b) showed that, at the concentration of 75 μg P/mL or lower, BPNPs caused no significant change in cell viability and the morphological appearance of RAW264.7 macrophages.

**Figure 7:**
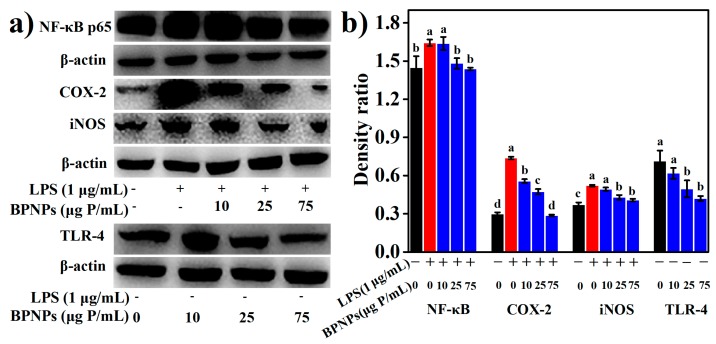
Effects of BPNPs on the levels of nuclear factor kappa beta (NF-κB) p65 (*n* = 3), cyclooxygenase-2 (COX-2) (*n* = 3), inducible NO synthase (iNOS) (*n* = 3), and Toll-like receptor 4 (TLR-4) (*n* = 3) in RAW264.7 cells with or without lipopolysaccharide (LPS) stimulation: (**a**) Western blotting analysis and (**b**) densitometry. Data were expressed as means ± standard deviations, with different lowercase letters marking significant differences (*p* < 0.05, ANOVA followed by Tukey’s HSD test).

LPS significantly increased the production of several proinflammatory mediators, including nitric oxide (NO), tumor necrosis factor-α (TNF-α), interleukin-6 (IL-6), and interleukin-1β (IL-1β) by the macrophages (*p* < 0.05) (Figure 6a–d), and these effects were markedly attenuated by the pretreatments with BPNPs for 6 h in a dose-dependent manner (*p* < 0.05). Prostaglandins, which are synthesized from arachidonate by the action of cyclooxygenase (COX) isoenzymes, play a key role in the development of cardinal signs of acute inflammation at the inflamed site [38]. LPS significantly upregulated the expression of COX-2 in RAW264.7 cells, as evaluated by Western blotting analysis (Figure 7a) and subsequent densitometry (Figure 7b) (*p* < 0.05), and this effect was prevented by the 6-h pretreatments with BPNPs, showing an evident dose-dependence. These results suggest that BPNPs could exert anti-inflammatory effects against intestinal inflammatory milieu.

The LPS-induced burst of NO production is likely the result of an increased expression of inducible NO synthase (iNOS) (Figure 7a,b). The 6-h pretreatment of RAW 264.7 cells with BPNPs at 25 μg P/mL or 75 μg P/mL significantly blocked the LPS-dependent increase in iNOS levels, suggesting that BPNPs attenuated NO production by downregulating the expression of iNOS in RAW264.7 cells. Nuclear factor kappa beta (NF-κB) is a key transcription factor governing COX-2 and iNOS expression as well as proinflammatory cytokine production, and was evidently activated by LPS stimulation for 1 h according to Western blotting (Figure 7a,b). The 6-h pretreatment of RAW 264.7 cells with BPNPs at 25 μg P/mL or 75 μg P/mL resulted in the significant inhibition of the LPS-dependent increase of NF-κB p65 protein (*p* < 0.05), suggesting that BPNPs exerted anti-inflammatory activities by blocking NF-κB activation in RAW264.7 cells.

LPS signals through the Toll-like receptor 4 (TLR4)/NF-κB pathway in macrophages. The results of Western blotting (Figure 7a,b) revealed that the treatment of RAW264.7 cells with 25 μg P/mL and 75 μg P/mL BPNPs for 6 h markedly decreased the levels of TLR4 protein (*p* < 0.05), suggesting the desensitization of mouse macrophages by these treatments. The endotoxin level in the stock solution of BPNPs (300 μg P/mL) was determined to be 0.042 EU/mL (equivalent to that of 0.014 ng/mL LPS), and according to a previous report by Fahmi et al. (1995) [39], an endotoxin level of 0.1 ng/mL LPS or lower was not sufficient to desensitize mouse macrophages. Thus, it seems that BPNPs could act on the LPS receptor TLR4 to exert their anti-inflammatory activities. Upon binding to LPS, TLR4 at the plasma membrane was found to be endocytosed and subsequently degraded in late endosomes/lysosomes as the rapid mechanism of LPS desensitization in human monocytes [40]. LPS stimulation has also been discovered to promptly accelerate the endocytic uptake of gold nanoparticles by RAW264.7 cells [41]. As reported by Tsai et al. (2012), the endocytic uptake of gold nanoparticles could induce the rapid translocation of cell surface TLR9 into the phagosomes in RAW264.7 cells [42]. Thus, we hypothesize that the cellular uptake BPNPs by macrophages induced an endocytic translocation and degradation of the cell surface of TLR4, leading to the blockage of the TLR4/NF-κB signaling pathway.

## 3. Conclusions

*Synechococcus* 7002 accumulate BPNPs within cells, maximumly at the late logarithmic growth phase when the medium contained 0.74 mM of KH_2_PO_4_, 11.76 mM of NaNO_3_ and 30.42 mM of Na_2_SO_4_, respectively. BPNPs in the cell extract of *Synechococcus* 7002 can be purified and further fractionated using Sephadex G-100 gel filtration and 100 kDa of ultrafiltration, respectively. BPNPs seem to survive the gastrointestinal digestion, and could attenuate the LPS-induced inflammatory responses in RAW264.7 cells via the suppression of the TLR4/NF-κB signaling pathway. Thus, there is promise in the use of BPNPs for the improvement of intestinal health. However, further studies are needed to evaluate their postbiotic efficacy in animal models.

## 4. Experimental Section

### 4.1. Chemicals

MTT, dimethylsulfoxide (DMSO), radioimmunoprecipitation assay (RIPA) lysis buffer (50 mM of Tris-HCl pH 7.4, 150 mM of NaCl, 1% Triton X-100, 1% sodium deoxycholate, and 0.1% SDS), DAPI, phenylmethanesulfonyl fluoride (PMSF), and Tris-buffered saline containing Tween 20 (TBST) (10 mM of tris (pH 7.5), 150 mM of NaCl, 0.05% Tween 20), pepsin, pancreatin, and bile salts were provided by Sigma-Aldrich Co. (Shanghai, China). Dulbecco’s modified Eagle’s medium (DMEM), the Pierce BCA protein assay kit, the enhanced chemiluminescence (ECL) detection kit, sandwich enzyme-linked immunosorbent assay (ELISA) kits for mouse IL-1β, IL-6, and TNF-α, Dulbecco’s phosphate-buffered saline (DPBS) and Hank’s balanced salt solution (HBSS) without phenol red were obtained from ThermoFisher Scientific (San Jose, CA, USA). Rabbit anti-mouse NF-kB p65 polyclonal antibody (ab32536), rabbit anti-mouse COX-2 polyclonal antibody (ab15191), horseradish peroxidase (HRP)-conjugated goat anti-rabbit IgG secondary antibody (ab205718), mouse monoclonal anti-β-actin antibody (ab6276), and HRP-conjugated goat anti-mouse immunoglobulin G (IgG) secondary antibody (ab6789) were purchased from Abcam (Shanghai, China). Mouse monoclonal anti-TLR4 antibody (sc-293072) was purchased from Santa Cruz Biotechnology (Shanghai, China). Rabbit monoclonal anti-iNOS antibody (13120S) was provided by Cell Signaling Technology (Shanghai, China). Fetal bovine serum (FBS) was purchased from ExCell Bio (Shanghai, China). A ToxinSensor™ Chromogenic LAL Endotoxin Assay Kit was purchased from GenScript Corporation (Nanjing, China). Other reagents used were of analytical grade and commercially available.

### 4.2. Cyanobacterial Strains and Culture Conditions

The *Synechococcus* 7002 strain was kindly provided by Prof. Jindong Zhao from the Institute of Hydrobiology, Chinese Academy of Sciences. *Synechococcus* 7002 was routinely maintained on agar plates of medium A at 30 °C under continuous illumination from cool white fluorescent lights (100 μmol photons m^−2^s^−1^) [43]. The inoculum was prepared by transferring one loop full of the organism from the agar plate to an Erlenmeyer flask (100 mL) containing 30 mL of medium A, and was incubated at 30 °C with shaking at 150 rpm under continuous illumination (100 μmol photons m^−2^s^−1^). The biomass was estimated by measuring the optical density at 750 nm using a BioTek PowerWave XS2 microplate spectrophotometer (BioTek Instruments, Inc., Winooski, VT, USA).

### 4.3. PolyP Measurements

All of the measurements were performed according Martin and Van Mooy (2013) with minor modifications [44]. Samples were made up to 0.5 mL with N-2-hydroxyethylpiperazine-N-2′-ethanesulfonic acid (HEPES) buffer (150 mM of KCl, 20 mM of HEPES-KOH, pH 7.0), stained with 60 µL of 100 µM of 4′,6-diamidino-2-phenylindole (DAPI, Sigma-Aldrich), vortexed, incubated for at least seven minutes, vortexed again, and measured in a final volume of 3 mL of HEPES buffer in a quartz fluorescence cuvette. Fluorescence at an excitation wavelength of 415 nm and an emission wavelength of 550 nm was measured on a HITACHI F-4600 spectrofluorometer (Hitachi High-Technologies Corporation, Tokyo, Japan). The bandwidths for excitation and emission were 5 nm and 10 nm, respectively.

### 4.4. Molybdenum Blue Assay for Orthophosphate

One mL of samples was mixed with 40 μL of ammonium molybdate solution (20 mM of ammonium molybdate in 5 M of sulfuric acid), followed by an addition of two drops of stannous chloride solution (2.5 g of SnCl_2_·2H_2_O dissolved in 100 mL of glycerine), and this mixture was incubated at 25 °C for at least five minutes. The orthophosphate concentration was determined by measuring the absorbance at 650 nm. NaH_2_PO_4_ was used as a standard, and a calibration curve was established for each set of samples.

### 4.5. Extraction and Purification of BPNPs

Cells were harvested by centrifugation at 6000× *g* for 10 min, resuspended in an equal volume of HEPES buffer, boiled for 20 min, and cooled immediately in an ice-water bath. After removal of cell debris by centrifugation at 10,000× *g* for 10 min, the supernatant was designated as the cell extract. BPNPs were purified from the cell extract by preparative gel chromatography using a water-equilibrated Sephadex G-100 (Pharmacia) column (2.8 cm × 50 cm). Before loading onto the column, the supernatant was filtered through 0.22-μm filters. The column was eluted with Milli-Q water at a flow rate of 1 mL/min. The eluent was monitored at 220 nm by a Shanghai HuXi analysis instrument HD-3 UV detector (Shanghai HuXi Analysis Instrument Factory Co., Ltd., Shanghai, China), and the peaks were collected manually. Ultrafiltration centrifuge tubes (Merck Millipore, Burlington, MA, USA) with a molecular weight cutoff of 100 kDa and 3 kDa were used to further fractionate and concentrate the fraction containing BPNPs. The endotoxin level in the stock solution of BPNPs was determined using an endotoxin detection kit according to the manufacturer’s instructions.

### 4.6. Electrophoretic Analysis of BPNPs

The electrophoresis of BPNPs in urea/polyacrylamide gels was performed according to Kumble and Kornberg (1995) with minor modifications [45]. Briefly, the urea/polyacrylamide gel was prepared by mixing 6.31 g of urea, 2.25 mL of acrylamide solution (38 g of acrylamide and 2 g of bisacrylamide dissolved in deionized water to a final volume of 100 mL), 3 mL of Tris borate buffer (450 mM, pH 8.3), 3 mL of 13.5 mM of EDTA, and deionized water to a final volume of 15 mL. After the addition of 2.5 mg of ammonium persulfate and 15 µL of *N*,*N*,*N*′,*N*′-tetramethylethylenediamine (TEMED), the gel was poured (83 mm × 95 mm × 1.5 mm) and allowed to polymerize. Samples were mixed with a 4-µL 5× sample buffer (50% sucrose, 0.125% bromphenol blue, 13.5 mM of EDTA and 450 mM of Tris borate at pH 8.3) and loaded on the gel. Electrophoresis was at 120 V until the bromphenol blue was 4 cm to 5 cm from the top of the gel. The gel was stained by agitation for 15 min in the fixative solution (0.05% toluidine blue, 25% methanol and 5% glycerol) followed by destaining in the same solution without toluidine blue.

### 4.7. Characterization of BPNPs

DLS and TEM were used to characterize BPNPs. The DLS analysis was performed on a Zetasizer Nano ZS 90 (Malvern Instruments, Herrenberg, Germany) equipped with a 633-nm He-Ne laser using a constant scattering angle of 90° at 25 ± 0.1 °C. To in situ visualize BPNPs within the cells of *Synechococcus* 7002, we fixed algae cells with 2.5% glutaraldehyde in 0.1 M of PBS (136.89 mM of NaCl, 2.67 mM of KCl, 8.1 mM of Na_2_HPO_4_, 1.76 mM of KH_2_PO_4_) for 2 h at 25 °C and for another 12 h in a 4 °C refrigerator, followed by staining with 1% OsO_4_ for 30 min, ultrathin sectioning and observation under a JEM-1200 electron microscope (JEOL, Tokyo, Japan) operating at 80 kV. For TEM measurements of purified BPNPs, sample solutions were dropped onto carbon-coated copper grids, air-dried, and examined in a JEM-1200 electron microscope at 80 kV.

### 4.8. Simulated GID

In vitro digestion was carried out by a slight modification of the method of Minekus et al. [46]. In detail, 40 μL of pepsin solution (100 mg/mL in 0.1 N of HCl, pH 2.0) was added to 2.0 mL of BPNPs (75 μg P/mL), and the pH was adjusted to 2.0 with 1 N of HCl, followed by incubation at 37 °C for 2 h. After the pH was adjusted to 6.5 with 1 N of NaHCO_3_, 160 μL of a mixture containing pancreatin (4 mg/mL) and bile extract (24 mg/mL) in 0.1 N of NaHCO_3_ was then added, followed by incubation at 37 °C for an additional 2 h. Orthophosphate and polyPs in the digestive fluid were determined using the molybdenum blue assay and DAPI fluorescence, respectively. BPNPs were fully hydrolyzed into orthophosphate by incubating in 2 N of HCl at 95 °C for 40 min.

### 4.9. Cellular Experiments

Murine macrophage RAW264.7 cells were originally obtained from the Cell Bank of the Chinese Academy of Sciences (Shanghai, China), and were maintained routinely in DMEM medium supplemented with 10% FBS, 100 units/mL of penicillin, and 100 μg/mL of streptomycin at 37 °C in a 5% CO_2_ atmosphere. RAW264.7 cells were used at passage levels of 10 to 20 and subcultured every three to four days. To evaluate cellular toxicity, cells were seeded at a density of 1 × 10^4^ cells/well in 96-well plates and cultured for 24 h in complete media. The cells were rinsed three times with DPBS, and incubated in complete media with and without BPNPs for another 24 h. Cell viability was measured by MTT assay. Briefly, cells were incubated with 1.2 µM of MTT in fresh medium for 4 h, and the resulted formazan crystals were dissolved with DMSO, followed by measuring the absorbance at 570 nm on a Synergy H4 hybrid microplate reader.

To assay the production of cytokines and nitric oxide (NO), RAW264.7 cells seeded into a 24-well plate (1.0 × 10^5^ per well) were cultured for 24 h in complete media. Cells were incubated in complete media with and without BPNPs for 6 h. Cells were rinsed with DPBS before the cells were incubated for 24 h in media supplemented with or without LPS (1 μg/mL). The cell culture supernatant was then harvested and stored at −20 °C before analysis.

Considering NO is extremely unstable and undergoes rapid oxidative degradation into nitrite and nitrate, NO production was assayed by measuring the nitrate and nitrite levels in cell culture supernatants using a Griess reagent, as described by Green et al. (1982) with minor modifications [47]. Briefly, the culture supernatant (100 μL) was mixed with an equal volume of freshly prepared Griess reagent (1% sulfanilamide, 0.1% *N*-1-naphthyl ethylenediamine, and 5% phosphoric acid) for 10 min in a 96-well plate, and the absorbance at 540 nm was then measured on a plate reader (Bio-Tek, Winooski, VT, USA). Nitrite concentration was calculated from a sodium nitrite standard curve. The concentrations of IL-1β, IL-6, and TNF-α in culture supernatants were determined using ELISA kits as per the manufacturer’s instructions.

For Western blotting experiments, RAW 264.7 cells were seeded into six-well plates (1.0 × 10^6^ per well) and were cultured for 48 h in complete media. The cells were rinsed and incubated with or without BPNPs for 6 h. For Western blotting analysis of TLR4, cells were rinsed and lysed in RIPA buffer containing PMSF (1 mM) and protease and phosphatase inhibitor cocktails at 4 °C for 30 min, and after centrifugation at 13,000× *g* for 15 min at 4 °C, the supernatant was used as a whole cell lysate for further analysis. For Western blotting analysis of NF-κB p65, COX-2, and iNOS, RAW 264.7 cells were rinsed and stimulated with or without LPS (1 μg/mL) for 1 h (NF-κB p65) or 24 h (COX-2 and iNOS). Cells were then lysed in RIPA buffer containing PMSF (1 mM) and protease and phosphatase inhibitor cocktails at 4 °C for 30 min, and after centrifugation at 13,000× *g* for 15 min at 4 °C, the supernatant was used as whole cell lysate for further analysis.

The protein concentration in whole cell lysate was determined with the Pierce bicinchoninic acid (BCA) protein assay kit following the manufacturer’s instructions. The cell lysate samples were denatured in SDS-loading buffer and separated by SDS-PAGE gel electrophoresis, followed by blotting onto a polyvinylidene fluoride (PVDF) membrane. The membrane was blocked with TBST containing 5% fat-free dried milk at 25 °C for 1 h, followed by incubation with anti-TLR4 (1:200 dilution), anti-NF-κB p65 (1:8000 dilution), anti-β-actin (1:5000 dilution), anti-COX-2 (1:500 dilution), or iNOS (1:200 dilution) overnight at 4 °C. The immune complexes were incubated with HRP-conjugated secondary antibodies (1:5000 dilution) at 25 °C for 1 h, and after being washed with TBST three times, they were visualized with an ECL detection kit on a Tanon-5200 Multi image analyzer (Tanon Science & Technology Co., Ltd., Shanghai, China). The band intensity was analyzed using Quantity One 4.6.2 software (Bio-Rad, Hercules, CA, USA).

### 4.10. Statistical Analysis

Statistical analyses were performed with SPSS software version 19.0 (SPSS, Inc., Chicago, IL, USA). Data were expressed as means ± standard deviations. The data were compared statistically by two paired Student’s t test or one-way analysis of variance (ANOVA) followed by Tukey’s HSD tests. Significant differences were defined at probability values (*p*-values) of *p* < 0.05, *p* < 0.01 and *p* < 0.001.
